# Supplementary material for: Inter-Laboratory Comparison of RT-PCR-Based Methods for the Detection of Tomato Brown Rugose Fruit Virus on Tomato
Source: Pathogens. 2022 Feb 3;11(2):207. doi: 10.3390/pathogens11020207 (PMC8877712; doi:10.3390/pathogens11020207)
Supplement: Supplementary file 1 [file pathogens-11-00207-s001.zip › pathogens-1533895-supplementary.pdf]

Supplementary Material

Supplementary material Table S1. List of Real Time RT-PCR kits and reagents used by the TPS participants and number of data set for each type. (*) highlights the recommended master mixes.

Real Time RT-PCR Kits	n° of data set
TaqMan® RNA-to-Ct™ 1-Step Kit (LifeTecnologies)*	14
iTaq One-Step Mastermix (Biorad)*	8
Luna® Universal Probe One-Step RT-qPCR Kit (NEB)	2
Ultraplex 1-Step Toughmix (Quantabio)	2
GoTaq Probe 1-Step RT-qPCR (Promega)	1
qScript™ XLT One-Step RT-qPCR ToughMix® (Quantabio)	1
AgPath ID one-step RT qPCR kit (Ambion)	1
qMAXSen™ One-Step Probe RT-qPCR Kit (CANVAX)	1
SS IV and Maxima Probe/ROX qPCR Master Mix (Thermo scientific)	1
CAPITAL™ qRT-PCR Probe Mix (Biotechrabbit)	1
TaqMan® Fast Virus 1-Step Master Mix (Applied Biosystem)	1
Reliance one-step Multiplex supermix (Biorad)	1

Supplementary material Table S2. Reproducibility values obtained for all the tests by each participant (considering valid data set). (–) = data set not provided.

	**L1**	**L2**	**L4**	**L5**	**L6**	**L7**	**L10**	**L11**	**L12**	**L14**	**L15**	**L16**	**L17**	**L18**	**L19**	**L20**
**ALK**	92%	92%	81%	90%	84%	84%	-	89%	-	92%	-	74%	78%	73%	92%	92%
**LOE**	77%	78%	81%	85%	83%	79%	-	83%	-	77%	-	80%	79%	70%	85%	83%
**ISH**	79%	76%	74%			66%	65%	73%			65%	70%	74%	68%	66%	78%
**M&W**	81%	77%	76%			69%	66%	80%			66%	71%	77%	76%	71%	78%
**PAN**	88%	74%	82%			88%	-	85%	82%	-	82%	75%	75%	84%	80%	88%
	**L21**	**L22**	**L23**	**L24**	**L25**	**L26**	**L27**	**L28**	**L29**	**L30**	**L31**	**L32**	**L33**	**L34**	**L35**	
**ALK**	84%	80%	92%	-		92%	92%		92%	92%	92%	90%	-	-	-	
**LOE**	85%	77%	81%	-		83%	83%		-	77%	85%	85%	-	-	-	
**ISH**	74%	74%	62%	79%	65%	71%	63%			71%	75%	78%	76%		74%	
**M&W**	81%	76%	71%	80%	66%	78%	65%			81%	75%	78%	77%	79%	81%	
**PAN**	75%	80%	83%	82%	71%	82%	69%	67%		88%		-	85%		-	

Supplementary material Table S3. List of TSP participants

**Partecipants**
AGROINNOVA - University of Torino – UNITO, Italy
Agroscope, Switzerland
ANSES - Laboratoire de la Santé des Végétaux – ANSES - LSV-UBVO, France
Bactochem, Israel
BASF Vegetable Seeds (BVS) – BASF, The Netherlands
BIOREBA AG, Switzerland
Centre for Research Experimentation and Training in Agriculture (CRSFA) "Basile Caramia" – CRSFA, Italy
Centro attività vivaistiche – CAV, Italy
Fera Science Limited, UK
Finnish Food Authority / Plant analytics / D120, Finland
Hazera Seeds - Hazera Health lab, Israel

Institute of plant protection - DLR Rheinpfalz, Germany
Instituto Nacional de Investigação Agrária, Plant Virology Laboratory – INIAV, Portugal
Instituto Nacional de Investigación y Tecnología Agraria y Alimentaria – INIA, Spain
Israel Plant Protection and Inspection services Virology Laboratory – PPIS, Israel
Julius Kühn-Institut, Inst. für Epidemiologie und Pathogendiagnostik - JKI-EPV, Germany
Laboratorio SFR Lombardia c/o Fondazione Minoprio, Italy
Leibniz Institute – DSMZ, Germany
LOEWE Biochemica GmbH – LOEWE, Germany
Main Inspectorate of Plant Health and Seed Inspection Central Laboratory – GIORIn, Poland
Microlab, Israel
Naktuinbouw, The Netherlands
National Institute of Biology, Department of Biotechnology and Systems Biology – NIB, Slovenia
Netherlands Food and Consumer Product Safety Authority (NVWA) National Reference Centre (NRC) - NVWA – NRC, The Netherlands
Österreichische Agentur für Gesundheit und Ernährungssicherheit, Abteilung für molekularbiologische Diagnose von Pflanzenkrankheiten – AGES, Austria
Phytopathology Laboratory, Agro-Bio Tech, University of Liège – ULG, Belgium
Plant Health and Environment Laboratory – PHEL, New Zealand
Plant Health and Microbiology Laboratory- PHML, Estonia
Plant Health Diagnostic National Reference Laboratory – PHDL, Hungary
Research Institute for Agriculture, Fisheries and Food – ILVO, Belgium
Scientia Terrae vzw – STRI, Belgium
University of Palermo, Plant Virology Lab, Italy
Ústřední kontrolní a zkušební ústav zemědělský – Ukzuz, the Czech Republic
Wageningen Plant Research – WPR, The Netherlands
